# Analysis of differences in pain and disability in people with adult scoliosis and nonspecific low back pain

**DOI:** 10.1186/1748-7161-8-S1-O11

**Published:** 2013-06-03

**Authors:** L Bissolotti, V Sani, M Gobbo, C Orizio

**Affiliations:** 1 Servizio di Recupero e Rieducazione Funzionale, Casa di Cura Domus Salutis, Brescia, Italy; 2 Sezione di Fisiologia Umana, Dipartimento di Scienze Biomediche e Biotecnologie, Università degli Studi di Brescia, Brescia, Italy; 3 LARIN: Laboratorio di Riabilitazione Neuromuscolare e Attività Fisica Adattata, Brescia, Italy

## Background

The cumulative effect of the aging process in patients with juvenile scoliosis and the appearance of new cases in adult scoliosis (AS)elicits great interest among clinicians and surgeons involved in management of spinal deformities[1]. Advances in scoliosis management are constantly required

## Aim

The aim of this paper is to compare pain, and disability, in patients with AS and nonspecific low back pain (NL).

## Methods

Cotrel method was used to assess Cobb angle (CA) on plain x-ray. Only patients with Cobb angle >15° were included in the study. Numeric Rating Scale (NRS, 0-10) was used to assess pain during last 48 hrs. Roland Morris Questionnaire (RMQ) and Oswestry Disability Index (ODI1.0) were used to evaluate disability.

## Results

AS-Group included 40 patients, 10 men and 30 women (age 61.8±11.5 years, BMI 23.6±2.8kg/m2). A single curve was present in 32 patients (80%). Primary curve averaged 27.1±11.5° (range, 15–63°), thoracic curve averaged 25.5±22.3° (range, 8–58°). NL-Group included 40 patients, 9 men and 31 women (age 58.2±10.9 years, BMI 23.9±3.2kg/m2). NRS score for AS-group was 5.9±1.8 (range, 2–10), while for NL group it was 5.1±2.2 (p>0.05). According to RMQ, the disability derived by low back pain presented a mean value of 11.3±4.4 points in AS-group (range, 2-22), while in NL-group it averaged 11.5±5.5 points (p>0.05). According to ODI1.0 AS-group presented a disability score of 33.9±17.6%, while in NL-group it was 32.6±18.8% (p>0.05). Sciatic pain was present in 27% of AS-Group and in 47% of NL-Group.

## Conclusions

As partially expected by the literature, the two examined groups did not present any relevant difference in terms of pain or disability [2]. Both groups presented a moderate level of pain according to NRS, and a moderate level of disability according either to ODI1.0 or RMQ. In this cohort of AS patients, conversely to the findings reported in literature, sciatic pain had a lower incidence than NL-group. Patients with AS, and a mild to moderate grade of spinal deformity, are not showing worse clinical features than patients affected by NL, and seem not to necessarily require more aggressive treatments than those usually adopted to contain disability in aging patients affected by common NL[3].
